# Tendon Transfer Procedures for Correction of Foot Drop Due to Injury to the Peripheral Nerves or Muscles

**DOI:** 10.1055/s-0044-1801322

**Published:** 2025-01-10

**Authors:** Henrik Lauer, Johannes Christoph Heinzel, Benedetta Vasselli, Farhad Farzaliyev, Jana Ritter, Jonas Kolbenschlag, Adrien Daigeler, Cosima Prahm

**Affiliations:** 1 Department of Hand, Plastic, Reconstructive and Burn Surgery, BG Klinik Tuebingen, University of Tübingen, Tübingen, Germany; 2 Department of Hand, Replantation and Microsurgery, BG Klinikum Unfallkrankenhaus Berlin, Germany; 3 Department of Hand, Replantation and Microsurgery, Charité University Medicine, Berlin, Germany

**Keywords:** foot drop, dorsiflexion, tendon transfer, peroneal nerve injury, tibialis posterior transfer

## Abstract

**Background** Foot drop is a challenging condition that significantly impacts the affected patient's mobility and quality of life. Tendon transfer has emerged as a viable treatment option. We hereby present data of the tendon transfer procedures in patients with foot drop in our department. Besides a detailed description of our surgical technique, we also compare our results with those reported in the literature.

**Methods** Data from 17 patients (11 males and 6 females) suffering from foot drop due to peripheral nerve or muscle lesions were retrospectively analyzed. All the patients underwent tendon transfer procedures between 2017 and 2022. Assessed outcomes encompassed parameters such as strength of foot dorsiflexion, the necessity for postoperative orthotic devices, and patient satisfaction. Demographic data, the time elapsed from injury/illness to surgery, and the underlying causes of foot drop were collected.

**Results** Postsurgery, 14 patients regained robust dorsiflexion strength (M4), while 2 exhibited slightly lower strength (M3) and 1 attained equal strength as on the unaffected side (M5). Corrective procedures were undertaken in five patients to address problems with tendon tension. After an average follow-up period of 11.9 months (± 9.13), 82.4% of patients reported a high level of satisfaction, although three patients experienced persistent foot inversion. Most patients (94.1%) no longer required orthotic devices following the operative procedure.

**Conclusion** A tendon transfer procedure for correction of foot drop has proven to be a safe and effective treatment option, resulting in a high level of patient satisfaction and restoration of quality of life.

## Introduction

Foot drop presents a significant impediment to walking and profoundly impacts any affected individual's quality of life. 1 The underlying causes of foot drop may vary, but they all share a common characteristic: a restriction in active dorsiflexion in the ankle joint. In the cases where active dorsiflexion is severely restricted, 2 3 4 restoring dorsiflexion strength is the main objective to restore the affected patients' quality of life.

Patients with paralysis of the tibialis anterior, extensor hallucis longus, and the extensor digitorum longus muscles may exhibit indirect compensatory mechanisms, leading to abnormal ranges of movement in the hip and knee joints while walking, a condition referred to as “steppage gait.” 5 6 Consequently, the demand for assistive devices like orthoses is high for patients with foot drop. 1

Addressing foot drop requires a multifaceted approach, as treatment possibilities are diverse. 7

Nonsurgical procedures include various approaches, such as the option of electrical stimulation. In cases of cerebral and neurogenic droop foot, it can improve the effective stride length during normal walk. 8 However, electrical stimulation has limitations when applied to patients with degenerated muscle fibers or direct muscle damage. Additionally, while robotic-assistive ankle–foot orthoses have proven to bring considerable functional improvement in patients with foot drop, it must be noted that the use of technological devices entails a certain dependence. 9

Ankle joint arthrodesis is a surgical option, although it entails complete loss of mobility in the ankle joint. 7 Other options are nerve reconstructions or transfers, but these are limited by time constraints, mechanism of injury, availability of the nerve stumps, and the affected nerve itself as was recently shown by Klifto et al. 7 Research conducted in a rat model could demonstrate that the regenerative capacity decreases significantly after only 3 months due to various changes in the distal stump. 10 Thus, the timing of nerve reconstruction is crucial after nerve injury for humans as well. 11 In contrast, tendon transfer procedures do not face the same temporal limitations. 12 Several techniques have been described so far, with the tibialis posterior transfer by means of transmembrane pull-through proving highly effective in terms of its biomechanical stability. 13 14 15 Additional tendon transfers, such as rerouting the peroneus longus in front of the lateral malleolus and securing it to the anterior tibial tendon (stirrup-plasty) can further increase stability by addressing the eversion deficit caused by palsy of the peroneus longus and brevis muscles. 16

The aim of this study was to contribute to the understanding of tendon transfer surgery by examining the etiology of foot drop, the reported postoperative necessity for orthopaedic devices, and patient satisfaction. It also focuses on outlining a detailed treatment algorithm for tendon transfer surgery in patients with foot drop. Integrating data and insights from retrospective analyses, this article will provide a clear guideline for physicians and surgeons to achieve the best outcomes for their patients when considering tendon transfer surgery for correction of foot drop.

## Material and Methods

In this analysis, retrospective data from patients who underwent a tendon transfer procedure for correction of foot drop in our hospital between January 2017 and December 2022 were included. The study protocol was approved beforehand by the University of Tübingen's ethical committee (project number: 451/2021BO2).

Prior to and after surgery, all patients underwent assessments to measure their active and passive ankle range of motion (ROM) and strength levels according to the Medical Research Council (MRC) scale. Additionally, the necessity of assistive orthotic devices both before and after the tendon transfer procedure, any preexisting medical conditions, and concomitant medications were documented. Furthermore, postoperative patient satisfaction with the surgical result was documented during subsequent evaluations by the following scale:

- *Dissatisfied:* The patient is dissatisfied with the surgical outcome and there are notable concerns about the results and impact on daily life.
- *Neutral:* The patient feels neither satisfied nor dissatisfied; there is a sense of indifference or ambivalence toward the surgical outcome.
- *Satisfied:* The patient is generally satisfied with the surgical outcome, experiencing improvements in the condition and overall well-being.
- *Very satisfied:* The patient is highly satisfied with the surgical outcome, perceiving substantial positive changes in health and quality of life.

To ensure comprehensive data collection, various demographic variables, including age, gender, underlying causes of foot drop, time elapsed from injury/onset of illness to the operation, and prior therapeutic interventions, were analyzed. Moreover, we closely observed and documented instances where revision surgeries were required after tendon transfer.

The inclusion criterion was a permanent foot drop or foot lifting weakness resulting from peripheral nerve or muscle lesions. Therefore, patients with paresis or palsy of the tibialis anterior and peroneal muscle group were included. The strength of the donor muscles (tibialis posterior muscle) had to be at least M4 according to the MRC scale.

**Fig. 1:**
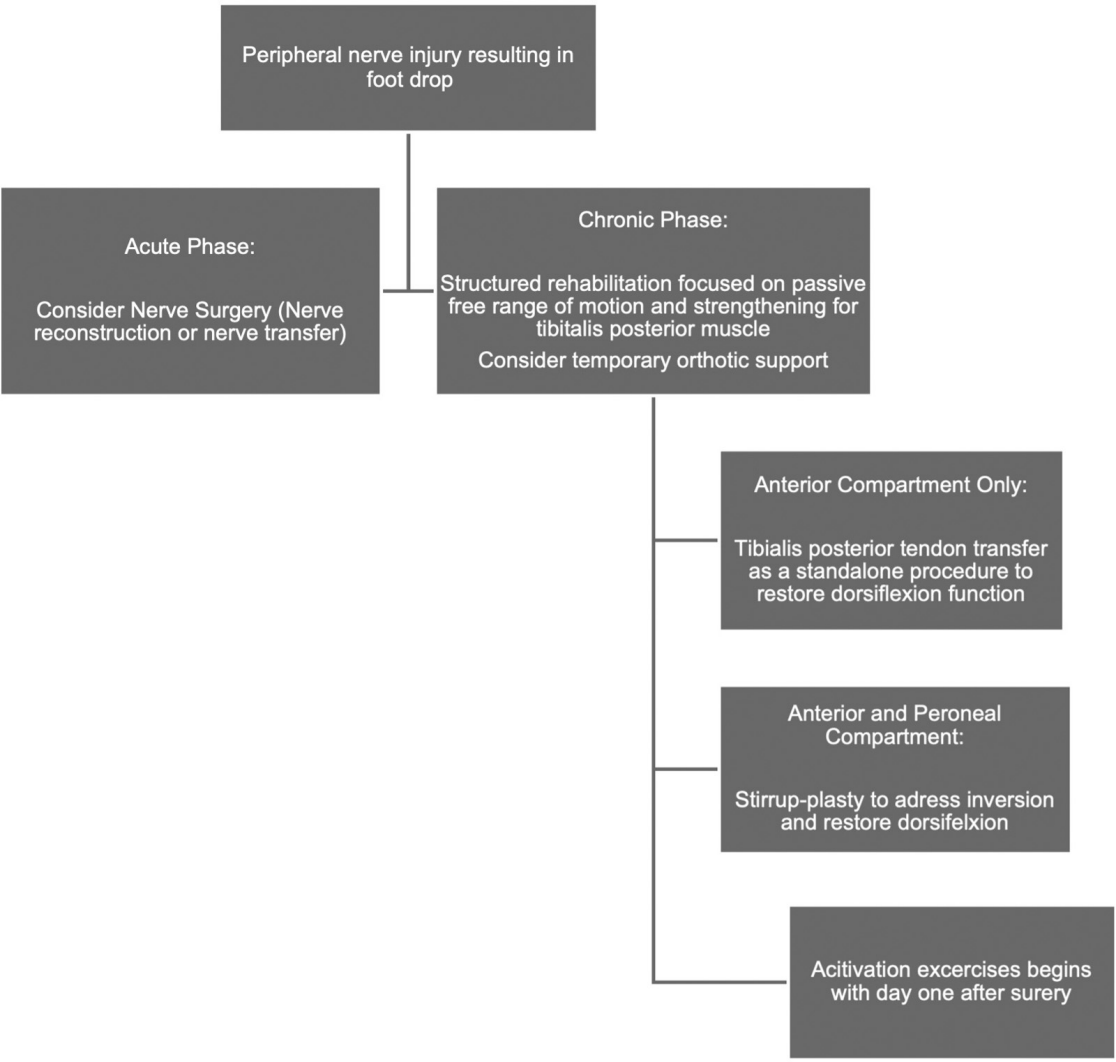
Treatment algorithm for peripheral nerve injuries resulting in foot drop. Individualized care and ongoing assessment are essential components of ensuring optimal outcomes for these patients.

The exclusion criteria encompassed (1) central lesions such as intracerebral or extracerebral tumors, cerebral ischemia, and multiple sclerosis and (2) peripheral lesions with chance of spontaneous nerve and muscle recovery as assessed and determined by a neurological examination ( Fig. 1 ).

### Surgical Technique

All surgical procedures were performed under general anesthesia with the patient in the supine position. We recommend applying a tourniquet to the thigh for dissection. The first incision is made over the distal lower leg in the course of the tibialis anterior tendon. Attention must be paid to the accompanying neurovascular bundle. A second incision is made at the level of the attachment point of the tibialis posterior muscle at the cuneiform bones for cutting the tendon. A third incision is made at the medial aspect of the lower leg. Through this incision, the pull-through of the dissected tendon through the interosseus membrane into the anterior compartment is performed. Therefore, the interosseous membrane must be longitudinally opened proximally over a distance of at least 5 cm. This allows us to achieve an optimal, that is, obtuse, angle, for the passage of the tendon. Subsequently, the tendon is sutured to the tibialis anterior tendon at the level of the ankle joint under passive dorsal extension using the technique known as side-to-side tenorrhaphy. 17 It is of paramount importance to assess the pretension under assistance and adjust it if necessary.

In the event of peroneus muscle failure, the peroneus longus tendon can be detached via a fourth incision at the tendinomuscular junction level and then sutured premalleolarly to the aforementioned tendon's coaptation sites. Following the completion of the procedure, the foot should be maintained in a passive state, nearly in a neutral position. After careful hemostasis and wound closure, an orthosis with an integrated vacuum cushion must be worn for a duration of 6 weeks. From the first postoperative day, activation exercises must be performed while the foot is still immobilized and accompanied by physical therapy.

## Results

A total of 17 patients (11 males and 6 females) were included in the retrospective review. The mean age of the patients was 36.3 (± 20.5) years. The average duration from the date of injury/initial onset of underlying illness to surgery was 53.7 (± 63.8) months. The average hospital stay was 3.7 (± 2) days.

**Fig. 2:**
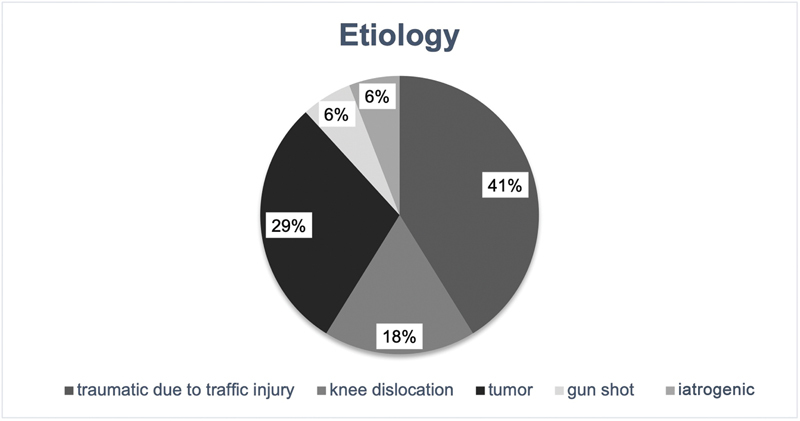
Etiology of foot drop undergoing tendon transfer procedure.

In 7 out of 17 patients (41.2%), foot drop was observable due to traumatic injuries stemming from high-velocity trauma associated with anterior compartment syndrome. In three cases, foot drop was a consequence of traumatic knee joint dislocation. In five cases, a tumorous mass involving the common peroneal nerve was observed, necessitating a planned resection involving the nerve. As a result of a hip joint surgery, one patient developed foot drop due to an iatrogenic injury to the sciatic nerve. Another patient was treated for the consequences of a gunshot wound, which resulted in partial injury to the sciatic nerve ( Fig. 2 ).

Coexisting conditions were mainly hypertension (3/17, 17.6%) and obesity (3/17, 17.6%). Diabetes and chronic pain syndrome was observed in one case (1/17, 5.9%).

**Fig. 3:**
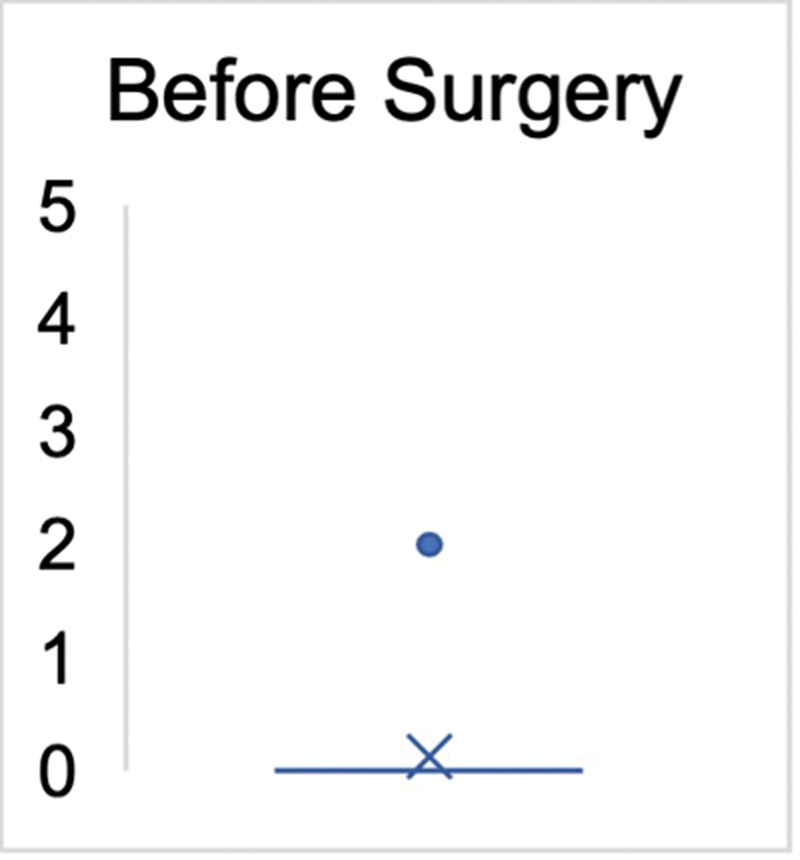
Medical Research Council (MRC) strength grade before surgery ( *n* = 17). The *cross* marks the mean value and the *dot* indicates the upper value ( *p* < 0.0001).

**Fig. 4:**
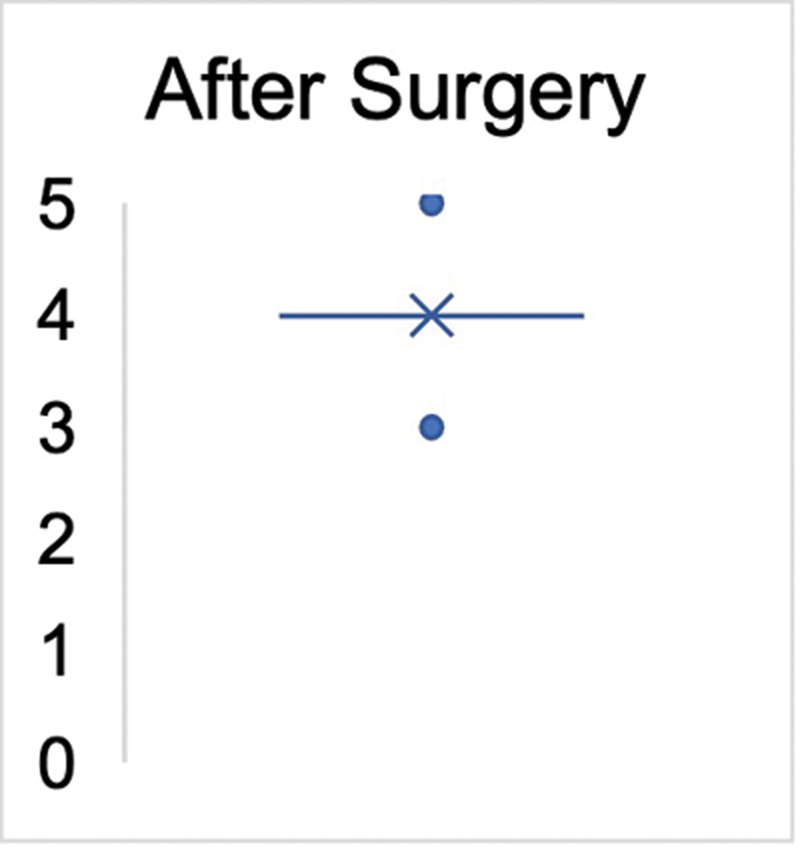
Significant difference in the Medical Research Council (MRC) strength grade after surgery ( *n* = 17). The *cross* marks the mean value and the *dots* indicate the upper and lower values ( *p* < 0.0001).

Prior to undergoing tendon transfer, all patients, except for one case (graded as M2), exhibited no signs of active dorsiflexion (graded as M0) in the ankle joint. In all patients, the ROM in the ankle joint allowed for at least 10 degrees of passive dorsiflexion. After the surgery (including revision surgeries), 15 patients achieved a robust dorsiflexion strength of M4 (88.2%). Two patients exhibited a strength of M3 (11.8%). One patient was documented with an M5 strength level (5.9%), indicating equal strength to the opposite side's dorsiflexion. Therefore, an average strength level of M4 (± 0.5) was achieved in all patients. The Mann–Whitney *U* test demonstrated statistical significance with *p* < 0.0001 ( Figs. 3 and 4 ).

**Fig. 5:**
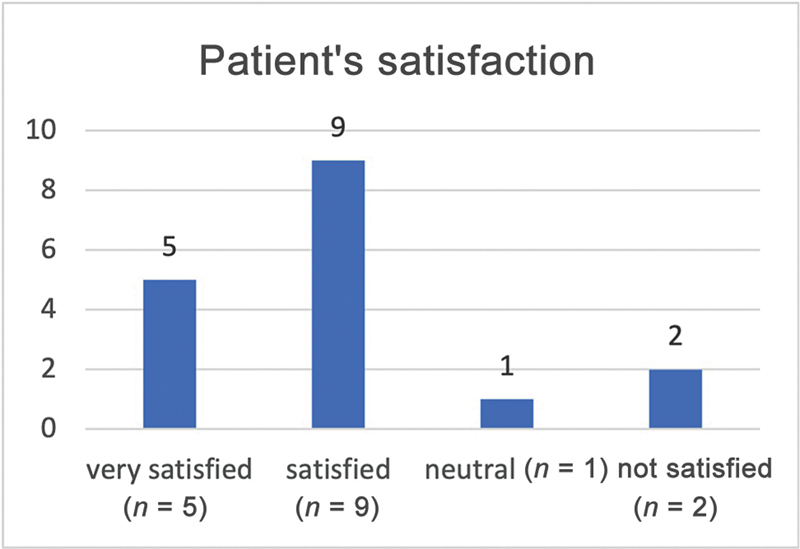
Patient's satisfaction after tendon transfer. Fourteen out of 17 patients were satisfied or very satisfied with the procedure.

In regard to ROM, all patients reached at least the neutral position, that is, 0 degrees, in the ankle joint during active dorsiflexion at the end of the respective follow-up period. The average follow-up period was 11.8 ± 9.1 months. At the end of the follow-up phase, 14 patients were “satisfied” or “very satisfied” (82.4%; Fig. 5 6 7 ), while 3 patients (17.6%) were bothered by the persistent inversion tendency of the foot. However, they declined further corrective interventions.

**Fig. 6:**
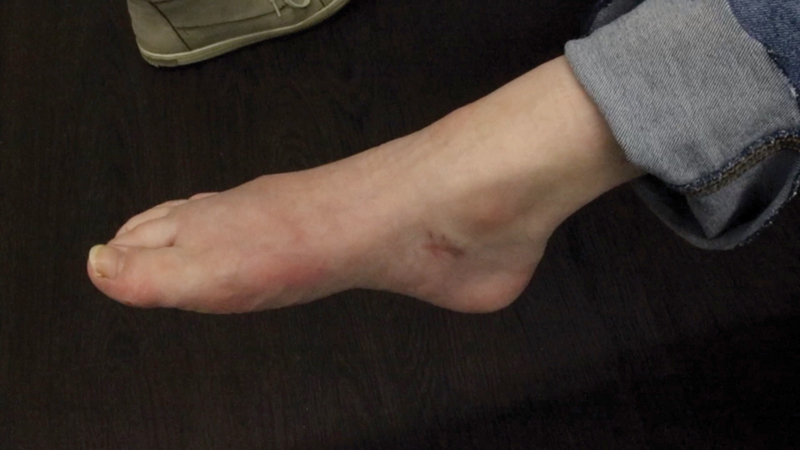
Active plantarflexion after tendon transfer.

**Fig. 7:**
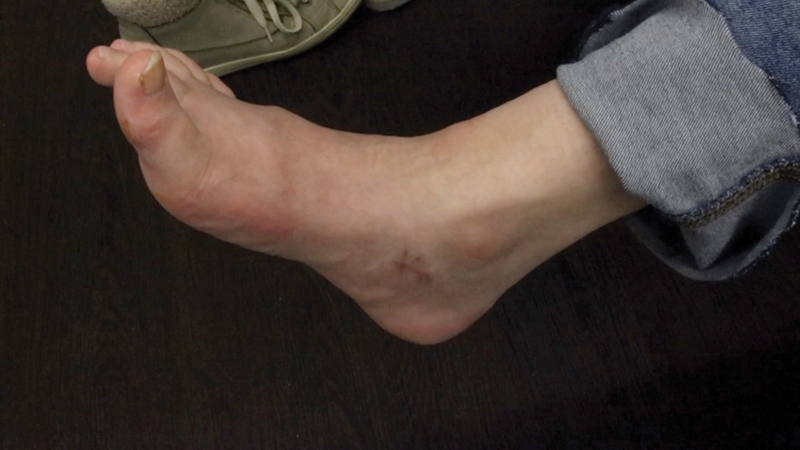
Active dorsiflexion after tendon transfer.

All patients were dependent on orthoses prior to the tendon transfer. Except for one patient (M4 in dorsiflexion), all patients were able to discontinue the use of orthoses after surgery. Subsequent revision surgery was performed on five patients (29.4%), with three exhibiting excessive inversion during active dorsiflexion and two showing insufficient dorsiflexion. One patient encountered a wound healing complication after surgery, which could successfully be treated by conservative measures ( Table 1 ).

**Table 1:** Summary of patient data

Gender	Age (during the surgery), y	Cause of foot drop	Time between injury and surgery (mo)	Hospital stay (d)	Coexisting conditions	Type of surgery	Degree of strength presurgery (MRC)	Degree of strength postsurgery (MRC)	Complications due to tendon transfer	Patient satisfaction	Revision surgery	Follow-up (mo)	Need for orthotic device after surgery
M	50	Traumatic injury/compartment-syndrome	240	10	None	Tibialis posterior transfer	M0	M4	None	Very satisfied	None	8	Not needed
M	17	Knee joint dislocation with affection of the communal peroneal nerve	17	3	None	Tibialis posterior and peroneus longus transfer	M0	M3	Insufficiency of tendon tension	Satisfied	Adjustment of the tendon transfer	8	Not needed
M	35	Knee joint dislocation with affection of the communal peroneal nerve	5	1	None	Tibialis posterior and peroneus longus transfer	M0	M4	None	Satisfied	None	7	Not needed
F	21	Iatrogenic lesion of the peroneal nerve	84	2	None	Tibialis posterior and peroneus longus transfer	M0	M4	None	Very satisfied	None	9	Not needed
F	62	Traumatic injury/compartment syndrome	8	3	Obesity, hypertension	Tibialis posterior transfer	M0	M5	None	Very satisfied	None	5	Not needed
M	50	Traumatic injury/affection of the sciatic nerve	8	3	None	Tibialis posterior and peroneus longus transfer	M0	M3	Wound healing disorder	Satisfied	None	6	Not needed
M	35	Traumatic injury/compartment syndrome	105	3	Obesity	Tibialis posterior transfer	M0	M4	None	Satisfied	None	7	Not needed
M	28	Gunshot with partial sciatic nerve injury	26	5	None	Tibialis posterior and peroneus longus transfer	M0	M4	None	Satisfied	None	8	Not needed
F	37	Traumatic injury/compartment syndrome	10	3	None	Tibialis posterior transfer	M0	M4	None	Very satisfied	None	8	Not needed
M	23	Knee joint dislocation with affection of the communal peroneal nerve	37	3	None	Tibialis posterior and peroneus longus transfer	M0	M4	None	Satisfied	None	8	Not needed
M	75	Traumatic injury/compartment syndrome	39	5	Hypertension, chronic pain syndrome	Tibialis posterior transfer	M0	M4	None	Satisfied	None	30	Not needed
M	30	Osteoblastic osteosarcoma affecting the peroneal nerve	36	4	None	Tibialis posterior and peroneus longus transfer	M2	M5	None	Very satisfied	None	8	Not needed
F	73	Monophasic fibrinous synovial sarcoma affecting the peroneal nerve	168	2	Diabetes, hypertension, obesity	Tibialis posterior and peroneus longus transfer	M0	M4	Insufficiency of tendon tension	Satisfied after revision surgery	Adjustment of the tendon transfer	6	Not needed
F	15	Neurofibromatosis with affection of the peroneal nerve	36	3	None	Tibialis posterior transfer and tenodesis of the extensor hallucis longus tendon	M0	M4	Insufficiency of tendon tension	Satisfied after revision surgery	Adjustment of the tendon transfer	24	Not needed
F	18	Iatrogenic sciatic nerve injury due to hip surgery	36	5	None	Tibialis posterior and peroneus longus transfer	M0	M4	Excessive inversion during dorsal extension	Neutral	Adjustment of the tendon transfer and additional transfer of the peroneus brevis tendon to the tibialis posterior/anterior tendon	18	Not needed
M	39	Traumatic injury/compartment syndrome	46	3	None	Tibialis posterior transfer	M0	M4	Slight inversion during dorsal extension	Not satisfied	None	7	Needed
M	9	Iatrogenic lesion of the peroneal nerve	12	4	None	Tibialis posterior and peroneus longus transfer, tenodesis of the toe extensors	M0	M4	Excessive inversion during dorsal extension	Not satisfied	Revision tenodesis	35	Not needed

Abbreviation: MRC, Medical Research Council.

## Discussion

In this retrospective chart review examining tendon transfer procedures for foot drop, we evaluated the ROM and strength grades as the main indicators of a successful surgery, alongside assessing patient satisfaction. Our findings provide insights into the outcomes of tendon transfer surgery, shedding light on the functional improvements achieved and the overall contentment reported by patients postoperatively.

The findings demonstrate a clear improvement in foot lifting strength and a notable reduction in the need for orthotic devices after the procedure.

Foot drop is a frequently overlooked symptom that imposes substantial restrictions on mobility and thus heavily impacts the patient's social life. 1 18 Damage to the peroneal nerve is considered the most common peripheral cause of foot drop. 3 In our patient population, anterior compartment syndrome also emerged as a prevalent causative factor. Prompt and emergency treatment of compartment syndrome remains paramount in preventing severe sequelae; however, a high number of cases are still diagnosed and treated too late, resulting in associated complication of foot drop. 19

Another subset of our patients (18%) suffered from foot drop because of knee dislocation. Existing literature has reported a risk of over 50% for common peroneal nerve palsy as debilitating complication of knee dislocations. 20 In 29.4% of cases, peroneal or sciatic nerve resection was necessary due to tumor resection. Especially within the context of knee joint procedures, resection of the peroneal nerve during tumor surgery is often unavoidable and must be considered in therapeutic planning. 21 All these cases involved permanent damage to the motor conduction pathway or the muscles responsible for elevating the foot.

In general, the initial approach for every patient with foot drop should involve exploring causal therapy options. The extent to which therapy is possible and useful depends on the subjective limitation, the site of damage, and the duration and severity of the paresis. 18 For peripheral damage, however, causal therapy is of limited value.

Klifto et al showed in a meta-analysis with more than 1,200 patients that neurolysis provided good functional results only for nerve entrapments. For all other damage patterns of the common peroneal nerve (transection/cut, avulsion/rupture), tendon transfer showed the best functional outcome with a significant difference compared to ankle–foot orthosis. Nerve reconstruction by nerve graft and neuromusculotendinous transfer also showed excellent function, but only for avulsion injuries of the peroneal nerve, and nerve reconstruction was not superior compared to tendon transfer. 7 22

An interesting finding of our study is that even long-standing foot drop can be corrected with a very good functional outcome following tendon transfers regarding strength, range of dorsiflexion, and patient satisfaction. For example, one patient included in the study (a 50-year-old) with 240 months of existing foot drop showed a strength of M4 in dorsiflexion and subjective high satisfaction as a result of tendon transfer. It appears that maintaining free passive joint mobility plays a pivotal role in achieving such positive outcomes. Physical therapy and wearing orthotics are very important after the occurrence of foot drop to avoid a pointed foot deformity. If the gastrocnemius and soleus muscles are structurally shortened, an achillotomy should be contemplated. Here, various surgical procedures are available, such as the percutaneous achillotomy as minimal invasive treatment. 23

Achieving strength equal to that of the unaffected contralateral side after tendon transfer procedures is not expected. In our patient population, only one patient showed a strength equal to the contralateral side, while 82.2% showed an MRC grade M4 strength. In contrast, Cho et al reported that the strength of foot elevation corresponds to only about one-third of the healthy side after tibialis posterior transfer. 24

In terms of the gait pattern, the crucial factor is not merely the strength but rather the ability to lift the foot against gravity, just as the different initial level of function does not necessarily correlate with the degree of severity of paralysis. 18 Moreover, the tenodesis effect is an advantage of tendon transfer so that even with insufficient active dorsiflexion, the foot experiences sufficient elevation during gait. 16 25

Further studies have shown that patient satisfaction was significantly increased after tendon transfer, the walking pattern showed a significant improvement, and long-term results indicated no higher-grade secondary problems. 16 24

The addition of the peroneus longus tendon transfer to the tibialis posterior transfer (stirrup-plasty) is indicated for a nerve damage that also involves the superficial peroneal nerve, either distally or at the level of the sciatic nerve. Stirrup-plasty provides bilateral stability against supinating or pronating forces. 16 However, the tension with which the tendon transfer must be applied is of crucial importance. The risk of persistent inversion tendency as well as insufficient dorsiflexion must be considered and communicated with the patient. Accordingly, corrective surgery with posttensioning was necessary and performed in 29.4% of patients. In any case, the treatment concept should be discussed with the patients to avoid unrealistic expectations.

The strongest argument for tendon transfer is the prospect of walking without orthotics. Over 94% of the patients included in our study were no longer dependent on an orthosis, a result that was also observed in similar studies. 16 26

### Limitations of the Study

The data presented in this study were evaluated through a retrospective and single-center investigation, thus limiting the sample size. The patient group included displays heterogeneity in terms of the various causes of foot drop, age, and the duration between initial onset and surgery.

The follow-up time is relatively short, spanning over approximately 11 months, and therefore does not allow any conclusions to be drawn about long-term follow-up issues such as arthrosis symptoms. The shortest time of follow-up was 5 months. However, dorsiflexion in the ankle joint to at least the neutral position with a strength level of M4, allowing for activities like walking and running without hindrance and no essential restrictions, was documented for most of the patients included in this study. Comparative studies with longer follow-up periods did not indicate more pronounced effects on secondary symptoms. 26 27 28 29 The risk of recurrence of foot drop following tendon transfer surgery, which can be caused by posterior muscle dysfunction, was not observed in this cohort or in other studies. 26 30

These outcomes underscore the effectiveness of tendon transfer as a safe and viable option in managing isolated foot drop, positively affecting patients' functional mobility and satisfaction. Collaborative efforts between orthopaedic surgeons, neurologists, physical therapists, and other health care professionals are vital in assessing patients comprehensively, tailoring treatment plans, and providing holistic support throughout the treatment journey. Additionally, investigating factors that may influence treatment success, such as patient-specific characteristics, lesion location, or preexisting medical conditions, can help identify optimal candidates for tendon transfer. Such personalized approaches to treatment can maximize the benefits and minimize potential risks, leading to more tailored and effective care.

## Conclusions

This study provides insights into the efficacy and impact of tendon transfer as a treatment modality for foot drop resulting from peripheral nerve or muscle lesions. The findings demonstrate an improvement in active ankle dorsiflexion and a notable reduction in the need for orthotic devices after the procedure, affirming the procedure's safety and effectiveness in managing foot drop.
